# Assessing the In Vitro Potential of Glatiramer Acetate (Copaxone^®^) as a Chemotherapeutic Candidate for the Treatment of *Cryptococcus neoformans* Infection

**DOI:** 10.3390/jof9080783

**Published:** 2023-07-25

**Authors:** Vinicius Alves, Pedro Henrique Martins, Bruna Miranda, Iara Bastos de Andrade, Luiza Pereira, Christina Takiya Maeda, Glauber Ribeiro de Sousa Araújo, Susana Frases

**Affiliations:** 1 Laboratório de Biofísica de Fungos, Instituto de Biofísica Carlos Chagas Filho, Universidade Federal do Rio de Janeiro, Rio de Janeiro 21941-902, Brazil; viniciusalves@biof.ufrj.br (V.A.); phmartinsdesouza@gmail.com (P.H.M.); brunamiranda@biof.ufrj.br (B.M.); iara.bastos@biof.ufrj.br (I.B.d.A.); luiza.giglio.pereira@gmail.com (L.P.); glauber@biof.ufrj.br (G.R.d.S.A.); 2 Laboratório de Fisiopatologia, Instituto de Biofísica Carlos Chagas Filho, Universidade Federal do Rio de Janeiro, Rio de Janeiro 21941-902, Brazil; takiyacm@biof.ufrj.br; 3 Rede Micologia RJ, FAPERJ, Rio de Janeiro 21941-902, Brazil

**Keywords:** *Cryptococcus neoformans*, cryptococcosis, drug repositioning, polysaccharide capsule

## Abstract

Cryptococcosis is a systemic mycosis affecting immunosuppressed individuals, caused by various *Cryptococcus* species. The current treatment utilizes a combination of antifungal drugs, but issues such as nephrotoxicity, restricted or limited availability in certain countries, and resistance limit their effectiveness. Repurposing approved drugs presents a viable strategy for developing new antifungal options. This study investigates the potential of glatiramer acetate (Copaxone^®^) as a chemotherapy candidate for *Cryptococcus neoformans* infection. Various techniques are employed to evaluate the effects of glatiramer acetate on the fungus, including microdilution, XTT analysis, electron and light microscopy, and physicochemical measurements. The results demonstrate that glatiramer acetate exhibits antifungal properties, with an IC_50_ of 0.470 mg/mL and a minimum inhibitory concentration (MIC) of 2.5 mg/mL. Furthermore, it promotes enhanced cell aggregation, facilitates biofilm formation, and increases the secretion of fungal polysaccharides. These findings indicate that glatiramer acetate not only shows an antifungal effect but also modulates the key virulence factor—the polysaccharide capsule. In summary, repurposing glatiramer acetate as a potential chemotherapy option offers new prospects for combating *C. neoformans* infection. It addresses the limitations associated with current antifungal therapies by providing an alternative treatment approach.

## 1. Introduction

Cryptococcosis is a systemic mycosis triggered by the *Cryptococcus* species and is contracted through the inhalation of infective particles in the form of spores or dried yeasts, usually present in soils, trees, and bird excreta [1]. After inhalation, *Cryptococcus* spp. survives in the lungs even though this organ is populated by specialized immune and defense cells such as alveolar macrophages, CD_4_ and CD_8_ T lymphocytes, and myeloid cells (such as dendritic cells), and despite the surface of the alveoli being covered with surfactants containing antimicrobial properties which maintains the tension lung surface [2,3]. After adaptation in the lung epithelium, these fungal cells can colonize the host’s respiratory tract for months or even years without causing any clinical symptoms [4]. The success of the infection and its dissemination throughout the body is linked to the ability of this fungus to evade the host’s immune system using several virulence factors such as increasing the polysaccharide capsule, increasing cell size (titan cells), melanin production, enzyme secretion, and others [3,5].

Since the installation of the infection is directly related to the evasion of the host’s immune system, immunocompromised individuals are more susceptible to this disease, namely, patients living with acquired immunodeficiency syndrome (AIDS), organ transplant recipients, and individuals under treatment with immunosuppressants and with hematological diseases [6]. *C. neoformans* develops a pathogenesis process in individuals who have a depleted immune system, but curiously, *C. gattii* manages to establish a pathogenesis in both immunocompromised and immunocompetent patients [4,7].

After establishment of the pulmonary infection, *Cryptococcus* spp. can reach the bloodstream, causing systemic conditions with damage to various anatomical sites such as the skin, eyes, prostate, and/or genitourinary tract. However, these species have a high affinity for central nervous system (CNS) colonization [8]. The preference for the brain is correlated with the fact that this organ has high concentrations of phenolic compounds, such as norepinephrine, serotonin, and dopamine, and these molecules are precursors for the activities of laccase enzymes that are involved in the synthesis of melanin [9,10,11]. The regions that can be primarily affected by *C. neoformans* infection, putamen, caudate nucleus (striate), and substantia nigra are the regions that have large amounts of dopamine receptors [10]. And indeed, this colonization gives rise to the most alarming manifestation of the disease, potentially leading to a life-threatening condition known as cryptococcal meningitis.

The treatment standardized by the World Health Organization (WHO) [12] for the treatment of cryptococcosis recommends the use of three antifungals: fluconazole (FLZ), amphotericin B (AmB), and flucytosine (5-FC) [12,13].

Some factors interfere with this treatment, justifying the search for effective alternatives. AmB is nephrotoxic and costly, and the application needs to be performed in a pre-hospital/hospital unit by a qualified health professional. 5-FC is not legally allowed in some countries, making it difficult to reach a consensus on the therapeutic scheme. Furthermore, FLZ, which, despite being the drug with the easiest access among those mentioned, presents patients with resistance in some regions of the planet. Added to the fact that no new class of antifungal drugs has been launched on the market for many years, the search for new therapeutic regimens for the treatment of cryptococcosis becomes necessary [14,15,16].

One approach to solving this problem is drug repositioning (DR). DR is based on the search for drugs that are already freely sold on the market and that can be redirected to another therapeutic activity [17]. As it is a drug that has already gone through the entire research and development process, the main advantage of DR is related to the reduction in time and investment. Drugs are repositioned because they already have all the pharmacokinetics and pharmacodynamics known, so these drugs can enter in the clinical phase trials more easily and quickly, as the risk of an unexpected adverse effect is minimal [18,19,20,21].

Although diseases caused by fungi are gaining more and more attention from the medical–scientific community, there is not enough investment in the development of new drugs that fight these infections. This concept, although in theory a recently discussed topic, has already been applied for some time in the search for new antifungals, such as studies with sertraline, statins, albendazole, ibuprofen, and others [22].

Glatiramer acetate (GA), also known as copolymer 1 (COP-1) or Copaxone^®^, is a synthetic polypeptide consisting of four naturally occurring and randomly arranged amino acids: L-glutamic acid (E), L-alanine (A), L-tyrosine (Y), and L-lysine (K), in the mean molar fraction of 0.141, 0.427, 0.095, and 0.338, respectively. The average molecular weight of this salt is between 5000 and 9000 Daltons (Da). This drug is found in the pharmaceutical form of subcutaneous injection, usually in concentrations of 20 mg/mL or 40 mg/mL, for daily applications. GA is a drug used primarily for the treatment of multiple sclerosis (MS), slowing the progression of the disease and reducing the rate of relapse [23,24].

Currently, in addition to MS, there are numerous other therapeutic applications for GA, including treatments for age-related macular degeneration [25], glaucoma [26], neurodegenerative diseases [27], ophthalmic diseases [28], Huntington’s disease [29], sclerosis amyotrophic lateral disease [30], Alzheimer’s disease [31], inflammatory bowel disease (IBD) [32], and microbicidal action on Gram-negative bacteria [33].

It is through the physical, chemical, and physiological characteristics of GA that the possibility of this drug being a candidate for pharmacological repositioning is analyzed. Its neuroprotective action, that is described as combating the progression of MS, becomes quite interesting since the most severe cases of cryptococcosis are characterized by cryptococcal meningitis [8,34]. GA possesses physicochemical properties that facilitate its traversal across the Blood–Brain Barrier (BBB), allowing it to reach the site of MS pathology, which could potentially overlap with the location where *Cryptococcus* spp. infections may manifest [34,35]. Despite extensive research on the applications of GA, none have specifically delved into its potential for treating fungal infections [34,35]. As much as there are many studies related to the use of GA, none of these applications have been investigated in fungal infections.

## 2. Materials and Methods

### 2.1. Strain

The species used was *Cryptococcus neoformans* var. *grubii* H99 (isolated clinic sample provided by Prof. doctor Arturo Casadevall at the Johns Hopkins Bloomberg School of Public Health, Baltimore, Maryland, USA). H99 is a wild-type strain, available from the American Type Culture Collection (ATCC) under catalog number 208821. The maintenance of the strain was carried out in a Sabouraud Dextrose Agar–SAB medium (Merck, Germany) and was grown for 2 days at 37 °C and stored at 4 °C.

### 2.2. Medium

#### 2.2.1. RPMI Medium

The RPMI medium for fungal cell cultivation was prepared with 10 g/L of RPMI 1640, with L-glutamine, and without sodium bicarbonate (Sigma Chemical Corporation, St. Louis, MO, USA) and was supplemented with 0.165 M of 3-morpholinopropane 1-sulfonic P.A.–MOPS (Vetec Química Fina Ltda., Rio de Janeiro, RJ, Brazil) and 0.01 M of glucose (Merck, Germany). After solubilization in ultrapure water, the pH was adjusted to 7.0 ± 0.2 at 25 °C, and the solution was filtered through a 0.22 µm pore membrane (JetBiofil, Guangzhou, China) and stored at 4 °C.

#### 2.2.2. Minimal Medium

The minimal medium (MM) that was used as a polysaccharide capsule inducer in *Cryptococcus* spp. through nutritional deprivation was prepared with 15 mM of glucose, 10 mM of MgSO_4_·7·H_2_O, 29 mM of KH_2_PO_4_, 13 mM of glycine, and 3 µM of thiamine, having been solubilized using ultrapure water, with the pH adjusted to 5.5 ± 0.2 at 25 °C and sterilized via autoclaving.

### 2.3. Antifungal Activity

To accurately evaluate the potential effects of drugs or chemical compounds on specific microorganisms, it is essential to establish specific parameters at the outset. These parameters involve identifying the most suitable test to be conducted in the chosen biological model and verifying that the composite being tested performs as expected [36]. In the case of studying the effects of antifungals on *Cryptococcus* spp., Ghannoum and colleagues [37] came to the conclusion that microdilution is the most appropriate test for evaluating the action of the drugs under study. This method was already utilized for assessing the effectiveness of all antifungal drugs used in the treatment of cryptococcosis. Through this analysis, it becomes possible to determine important measures such as the IC_50_ (half-maximal inhibitory concentration) and the minimum inhibitory concentration (MIC), both of which play a critical role in assessing the drug’s potential as a candidate for antifungal treatment.

The protocol used followed the determination of the European Committee on Antimicrobial Susceptibility Testing (EUCAST) [38]. For this, *C. neoformans* was cultivated in a solid Sabouraud medium for 24 h at 37 °C. After this time, five colonies were suspended in sterile water and homogenized for 15 s in a vortex mixer. With the help of a Neubauer chamber, the cells were counted so that it was possible to have an inoculum of 5 × 10^3^ cells/mL, in the RPMI medium for fungi, per well. Subsequently, in a 96-well plate, serial dilutions [*v*/*v*] were made with 40 mg/mL of glatiramer acetate (GA) (Copaxone^®^, Ivax pharmaceuticals, Runcorn, UK) in the RPMI medium for fungi, so that in the final volume of 200 µL, it was possible to obtain the concentrations from 10 mg/mL; 5mg/mL; 2.5mg/mL; 1.250mg/mL; 0.625 mg/mL; 0.312 mg/mL; 0.156 mg/mL; 0.078 mg/mL; and 0.039 mg/mL. For this, 100 µL of each concentration of GA was placed in triplicate and was concentrated twice, followed by 100 µL of the solution containing the fungal cells also being concentrated twice, totaling a final volume of 200 µL per well with the desired concentrations of both the drug, 10 mg/mL to 0.039 mg/mL, and the fungal cell, 5 × 10^3^ cells/mL, in each well. As a negative control, 200 µL of the RPMI medium containing the concentrations of GA without the fungal cells was used, and as a positive control, only 200 µL of the RPMI medium containing the stipulated concentration of *C. neoformans* cells was used. After growth at 35 °C for 72 h without agitation, this assay was analyzed in a microplate spectrophotometer at a wavelength of 530 nm, with only the RPMI medium and the respective concentrations of the drug without yeast as a blank. With this result, it was possible to calculate the IC_50_, the concentration responsible for inhibiting 50% of cell growth, and the minimum inhibitory concentration (MIC), the lowest concentration at which there was no more cell growth.

### 2.4. Cell Viability

The yeasts were centrifuged at 6708× *g* for 5 min, discarding the supernatant and washing them three times in PBS (pH 7.2 ± 0.2 at 25 °C). Cells were counted in a Neubauer chamber and normalized to 5 × 10^3^ cells/mL in RPMI and cultured at 37 °C for 7 days in a 12-well plate with a final volume of 1.5 mL, in the absence or in the presence of increasing concentrations (0.156 mg/mL to 1.2 mg/mL) of glatiramer acetate. As a negative control, 1.5 mL of the RPMI medium containing the highest concentrations of GA without the fungal cells was used, and as a positive control, only 1.5 mL of the RPMI medium containing the stipulated concentration of *C. neoformans* cells was used. Subsequently, 1 mL of cell suspension was collected from each well at 6708× *g* for 5 min and washed twice with PBS to remove drug residues in the supernatant. After meticulous washing, the pellet was resuspended in 1 mL of RPMI where 100 μL of the cell suspension were added, in triplicate, to a plate of 96 wells, after which 70 μL of a 2,3-bis-(2-methoxy-4-nitro-5-sulfophenyl)-2H-tetrazolium-5-carboxanilide (XTT) (Thermo Fisher Scientific, Waltham, MA, USA) was added and incubated for 4 h at 37 °C. Readings were taken past the necessary time in a microplate spectrophotometer with wavelengths of 450 nm and 660 nm. The XTT technique for assessing cell viability is a colorimetry based on the reduction in 2,3-bis-(2-methoxy-4-nitro-5-sulfophenyl)-2H-tetrazolium-5-carboxanilide) (XTT) to a soluble orange formazan compound, through NADH produced for mitochondrial membrane electron transport and an electron coupler. The analysis of the specificity of XTT absorbance was performed using the following equation as per recommended by the manufacturer [39].

### 2.5. Morphometric Analysis

The cells were grown in 15 mL of the liquid SAB medium at 37 °C under agitation with a constant at 5× *g* (~100 rpm–nominal). Subsequently, the yeasts were centrifuged at 6708× *g* for 5 min, discarding the supernatant and washing them three times with PBS (pH 7.2 ± 0.2 to 25 °C). Cells were resuspended in 1 mL of the RPMI medium for fungi and counted in a Neubauer chamber to standardize the inoculum at 5 × 10^3^ cells/mL. Cultures were maintained at 37 °C for 7 days in a 12-well plate with a final volume of 3 mL in the absence and presence of different concentrations of glatiramer acetate (0.625 mg/mL; 0.470 mg/mL; and 0.312 mg/mL), with RPMI as both the negative control and the medium with the highest drug concentration. To visualize the yeast under study, 1 mL of each culture condition studied was centrifuged at 6708× *g* for 5 min and washed once with PBS (pH 7.2 ± 0.2 to 25 °C). To carry out the negative contrast using India ink, the montage of the slide was given with 5 µL of the solution containing the fungal cells and 5 µL containing India ink, with both being visualized in the optical microscope Carl Zeiss™ AxioLab™ A1, (Carl Zeiss Microscopy GmbH, Jena, Germany). Random images of at least 50 cells were measured using the software ImageJ 1.8.0 (National Institutes of Health (NIH), Bethesda, MD, USA) [40] to measure the capsular and cell body diameter.

### 2.6. Immunofluorescence

After the different experimental conditions, aliquots containing 1 mL of each culture were centrifuged at 6708× *g* for 5 min and the cells were washed twice with PBS (pH 7.2 ± 0.2 at 25 °C) to remove the drugs present in the medium. After these procedures, cells were fixed with 4% paraformaldehyde (Sigma Aldrich, St. Louis, MO, USA) in PBS for 20 min. Subsequently, the cells were fixed, and the yeasts were washed twice in PBS and incubated with 1% albumin of bovine serum (BSA) [W/V] (Sigma Aldrich, St. Louis, Missouri, USA) in PBS (PBS-BSA) for 1 h at room temperature. Cells were washed again and incubated for 1 h at room temperature in the presence of the monoclonal antibody (mAb) 18B7 (10 μg/mL), and a mouse IgG1 with affinity for GXM of different cryptococcal serotypes [41,42]. Subsequently, the cells were centrifuged at 6708× *g* for 10 min, washed twice with PBS, and incubated for 10 min at room temperature with the cell wall dye Uvitex 2B (Polyscience Inc, Warrington, PA, USA) at a 1:1000 dilution in PBS [V:V]. Uvitex 2B binds to chitin (polymer of *N*-acetyl-*D*-Glucosamine) present in fungal cell walls. After incubation with the dye, the yeasts were washed four times with PBS to completely remove the unbound dye residue. Finally, cells were incubated with 10 μL/mL (1:100 in PBS) of the antibody secondary anti-mouse IgG Alexa^®^ 546 (ThermoFisher, Waltham, MA, USA) for 60 min at 37 °C. At the end of the incubation, the cells were centrifuged and washed three times with PBS at 6708× *g* for 10 min. A smooth blade (26 × 76 mm) was mounted by applying 10 µL of the processed cells onto it, which was then covered with a 20 × 20 mm cover slip from the manufacturer Waldemar Knittel Glasbearbeitungs GmbH (Varrentrappstr, Braunschweig, Germany). The resulting slide was observed using an Axio Observer fluorescence optical microscope (Carl Zeiss Microscopy GmbH, Jena, Germany).

### 2.7. Scanning Electron Microscopy

Cells under the culture conditions of interest were centrifuged at 6708× *g* for 5 min and washed three times in PBS (pH 7.2 ± 0.2 at 25 °C). Subsequently, the yeasts were fixed in 2.5% type I glutaraldehyde (Electron Microscopy Sciences, Hatfield, PA, USA) in a sodium cacodylate buffer (Electron Microscopy Sciences, Hatfield, PA, USA) of 0.1 M pH (pH 7.2 ± 0.2 at 25 °C) for 1 h in a room temperature environment. Then, the cells were centrifuged under the same conditions as mentioned above and washed with a post-fixation solution composed of a 0.1 M sodium cacodylate buffer; 0.2 M of sucrose, and 2 mM of MgCl_2_ (all compounds from Merck Millipore, Burlington, MA, USA). After post-fixation, the yeasts were placed on circulars coverslips #12 (Waldemar Knittel Glasbearbeitungs GmbH, Varrentrappstr, Braunschweig, Germany) for 20 min, which were previously treated with 0.01% poly-L-lysine (Sigma-Aldrich, MA, USA). The cells that had already adhered were dehydrated in increasing ethanol solutions (Merck Millipore, Burlington, MA, USA) and in ultrapure water [V:V] (30, 50, and 70% for 5 min, and two times at 95 and 100% for 10 min). After dehydration, the samples were dried using the critical point method (EM DPC 300, Leica Microsystems Europe), mounted on stubs (metallic support), and coated with a layer of gold/palladium (Au/Pd) using a metallizer (Balzers Union FL-9496, Balzers, Liechtenstein). The visualization was performed using a Tescan VEGA 3 LMU scanning electron microscope (Hitachi, Tokyo, Japan).

### 2.8. Extraction and Concentration of Secreted Polysaccharides

For the extraction of secreted polysaccharides (PS), cells were grown in 15 mL of the liquid SAB medium on a rotary shaker for 24 h at 37 °C and 5 × 10^5^ cells/mL of *C. neoformans* were inoculated in 250 mL of minimal medium in the absence or presence of different concentrations of glatiramer acetate, previously determined in previous results (0.625 mg/mL; 0.470 mg/mL; and 0.312 mg/mL). After 7 days under constant agitation at 37 °C, the cultures were centrifuged at 1677 g per 15 min to separate the cells from the supernatant. An aliquot was withdrawn from the cells for morphological analysis and the physical–chemical properties, and the obtained supernatant, which contains all the secreted polysaccharides was concentrated through ultrafiltration in the Amicon^®^ Purification System (Millipore, Danvers, MA, USA) using a membrane that retains any structure with a molecular mass equal to or greater than 10 kDa. After obtainment, the PS were stored at 4 °C for later analysis.

### 2.9. Zeta Potential (ζ) and Conductance

The Zeta potential (ζ) reflects the surface charge (in millivolts) of a given particle, such as cells and/or polysaccharides, that were interfaced with a liquid and may have its results altered by several factors such as ionic constitution of the medium dispersant, ionizable functional groups of the particle, or the adsorption of ions from the solution on the surface of the particle. The Zeta potential (ζ) was derived from the equation ζ = (4 Π η *m*)/*D*, where *D* is the dielectric constant of the medium, η is the viscosity, and *m* is the mobility particle electrophoresis [43]. Initially, 1 mL of the cells of interest was centrifuged at 6708× *g* for 5 min and resuspended in apyrogenic water to remove all the media present, as it may contain several ions that interfere with the surface charge analysis, altering the measures. An amount of 100 µL of cells from each condition studied was added to 1400 µL of apyrogenic water in a cuvette suitable for measuring the Zeta potential and conductance in the NanoBrook Omni equipment (Brookhaven Instruments Corp., Holtsville, NY, USA). The measurement of the Zeta potential (ζ) of the isolated polysaccharides was carried out in a 10 mg/mL solution in apyrogenic water, as mentioned above.

### 2.10. Dynamic Light Scattering (DLS)

DLS is a technique that allows us to calculate the effective diameter and the polydispersion of molecules and particles. The Brownian motion of the sample in the liquid phase causes the laser focused on the sample to be scattered with different intensities, and from the analysis of these intensity fluctuations, it was possible to obtain the speed of the Brownian motion, with which the aid of the Stokes–Einstein relation allows us to determine the size of each molecule or particle [44,45]. A solution containing 10 mg/mL of PS in deionized water, of the different strains and study conditions, was prepared with the purpose of measuring the effective diameter of polysaccharide fibers. Measurements were performed using the NanoBrook Omni (Brookhaven Instruments Corp., Holtsville, NY, USA).

### 2.11. Passive Microrheology

Microrheology is a technique that uses probes on the micro/nano scale for the study of viscoelastic properties, such as viscous modulus (G’’), elastic (G’), and complex viscosity (η*). In passive microrheology, the inherent thermal energy of the sample was used to move the probes and obtain viscoelastic analyses [46,47]. The technique has, as its focus, the characterization of fluid complexes such as *C. neoformans* PS [48]. In an appropriate cuvette, 0.5 µL of microspheres (probes) was added to 1 µm of polystyrene (Polysciences, Inc., Warrington, PA, USA) and 2 mL of the PS sample to be analyzed at a pre-established concentration of 10 mg/mL. A method to control apyrogenic water was used, whose physicochemical properties were already well known. Measurements were performed using the NanoBrook Omni (Brookhaven Instruments Corp., Holtsville, NY, USA).

### 2.12. Statistical Treatment

All data were subjected to statistical analysis using the Prism 9.5 for MacOS (GraphPad Software, San Diego, CA, USA, https://www.graphpad.com/, accessed on 24 June 2023). *p* values for multiple comparisons were calculated via analysis of variance (ANOVA) and adjusted using Tukey’s multiple comparison test, where *p* < 0.05 was considered significant.

## 3. Results

### 3.1. The Antifungal Potential of Glatiramer Acetate in C. neoformans

**Figure 1:**
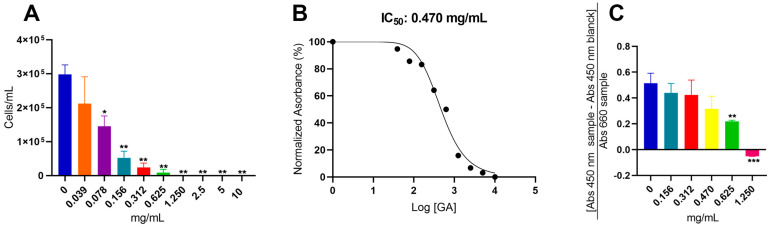
Influence of GA on cell proliferation and viability of *C. neoformans*. (**A**) GA microdilution assay from concentrations of 10 mg/mL to 0.03 mg/mL, (**B**) determination of IC_50_ of GA in *C. neoformans*, and (**C**) cell viability of *C. neoformans* in different concentrations of GA. Statistical treatment using the Student’s *t*-test * *p*-values < 0.05, ** *p*-values < 0.01. *** *p*-values < 0.001.

The initial study conducted to investigate the antifungal activity of GA involved a microdilution assay with decreasing concentrations of the drug (10 mg/mL, 5 mg/mL, 2.5 mg/mL, 1.250 mg/mL, 0.625 mg/mL, 0.312 mg/mL, 0.156 mg/mL, 0.078 mg/mL, and 0.039 mg/mL). This qualitative analysis aimed to determine the susceptibility of *C. neoformans* cells to GA. The minimum inhibitory concentration (MIC), which represents the lowest concentration of the drug inhibiting fungal cell growth [49], was determined to be 2.5 mg/mL for GA in *C. neoformans cells* (Figure 1A), based on the microdilution assay. Following the macroscopic analysis, absorbance measurements were taken to generate a curve correlating optical density with drug concentration. This curve allowed for the determination of the concentration at which 50% of cell growth was inhibited (IC_50_). As depicted in Figure 1B, the experimentally calculated IC_50_, obtained through triplicate experiments and samples, was determined to be 0.470 mg/mL of GA. This quantitative data is of the utmost importance as it guided the subsequent experiments investigating the cellular-level effects of GA.

Another critical aspect that was addressed was the metabolic activity (viability) of *C. neoformans* cells across various concentrations of GA. This differentiation aids in assessing whether GA exhibits fungistatic and/or fungicidal effects. For this purpose, five conditions were selected based on previous results: IC_50_ concentration (0.470 mg/mL), two concentrations below (0.156 mg/mL and 0.312 mg/mL), and two concentrations above (0.625 mg/mL and 1.250 mg/mL). As illustrated in Figure 1C, among the conditions studied, the concentrations of 1.250 mg/mL and 0.625 mg/mL of GA displayed a significant decrease in cell viability (*p*-values < 0.001 and 0.01, respectively; Student’s *t*-test). Conversely, the concentrations of 0.470 mg/mL (IC_50_), 0.312 mg/mL, and 0.156 mg/mL did not exhibit differences compared to the drug-free control. It is noteworthy that the negative value observed at a concentration of 1.250 mg/mL can be attributed to the theoretical formulation provided by the manufacturer (Equation (1)) [39]. Due to the growth reduction observed at this concentration, the absorbance readings of the samples were notably low, leading to negative results.

(1) $$Specific\;absorbance=\frac{\left[Abs\;450\;\mathrm{nm}\;\left(sample\right)-Abs\;450\;\mathrm{nm}\;\left(blanck\right)\right]}{Abs\;660\;\mathrm{nm}\;\left(sample\right)}$$

### 3.2. Effect of Glatiramer Acetate on the Structural and Physical–Chemical Properties of C. neoformans Cells

By employing these experimental procedures and analyzing the obtained data, valuable insights were gained regarding the antifungal activity of GA against *C. neoformans*. These findings provided the foundation for subsequent experiments investigating the effects of GA at the cellular level. After the experiments demonstrated that GA has antifungal potential and influences yeast cell metabolism, a series of other studies were carried out to investigate whether there is any change both in the structural properties of the polysaccharide capsule and in the properties of the physicochemical characteristics of *C. neoformans* cells. According to previous results, three concentrations were chosen for the performance of the studies in question: the IC_50_ (0.470 mg/mL), a concentration above (0.625 mg/mL), and one below (0.312 mg/mL).

**Figure 2:**
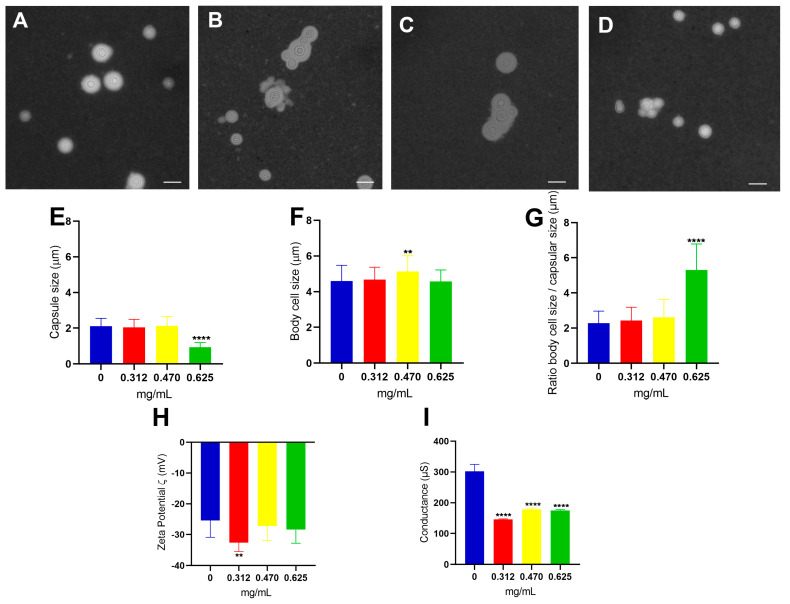
Morphological and physical–chemical analyses of *C. neoformans* cells under different concentrations of glatiramer acetate. Negative staining of *C. neoformans* without drug (**A**), under 0. 312 mg/mL (**B**), 0.470 mg/mL (**C**), and 0.625 mg/mL (**D**) of GA. Scale bar: 10 µm. Measurement of polysaccharide capsule size (**E**), cell body (**F**), ratio of cell size to capsular (**G**), Zeta potential (**H**), and conductance (**I**). Statistical treatment using the Student’s *t*-test ** *p*-value < 0.01. **** *p*-value < 0.0001.

A morphometrical analysis using brightfield light microscopy allowed us to observe that in the control (Figure 2A), fungal cells were dispersed without evidence of cell clusters. In contrast, yeasts in the presence of all concentrations of GA tend to aggregate, even at the lowest concentration used (0.312 mg/mL) (Figure 2B). Thus, most of the cells aggregated to each other, indicating that there was possibly a formation of biofilm, and the same could be observed in the IC_50_ (Figure 2C) and in the highest concentration (Figure 2D). With the images obtained from optical microscopy, it was possible to measure important parameters in the study of *Cryptococcus* spp.: the polysaccharide capsule, the cell body, and consequently, the ratio between them [50]. After evaluating the morphometric and statistical analyses of yeasts cultivated in the presence of GA in different concentrations, it was possible to show that there was a significant reduction in capsular size (*p*-value < 0.0001, Student’s *t*-test) at the highest concentration of GA (Figure 2E) and an increase in cell body size only for the concentration IC_50_ (*p*-value < 0.01, Student’s *t*-test) (Figure 2F). When we analyzed the relationship of cell/capsular size (Figure 2G), it was observed that the proportion of cell size to polysaccharide capsule size remained the same when compared to the control in almost all concentrations, except for the 0.625 mg/mL in which the cell development was of equivalent size but showed a decrease in the polysaccharide capsule. Taken together, the results indicate that GA can negatively modulate the capsule building process at the highest concentration used.

Another widely evaluated parameter in studies with *Cryptococcus* cells is the relationship of the cell surface's electronegativity and species pathogenesis. Glucuronic acid is the component of the polysaccharide capsule that was responsible for this load effective negative, and the increase or decrease in the amount of this sugar is directly correlated with virulent strains [51]. It is through the Zeta potential that it was possible to measure the charge difference between the cell surface and the medium in which it was embedded. The cells in the concentration of 0.312 mg/mL of GA showed a significant increase in the Zeta potential when compared to the drug-free control (*p*-value < 0.01, Student’s *t*-test), showing to be more electronegative than the other conditions (Figure 2H). The result of conductance allows us to observe that there is a decrease statistic (*p*-value < 0.0001, Student’s *t*-test) in all conditions where the cells grew in the presence of the drug (Figure 2I), showing that there is a decrease of the ionic charges present under these conditions.

**Figure 3:**
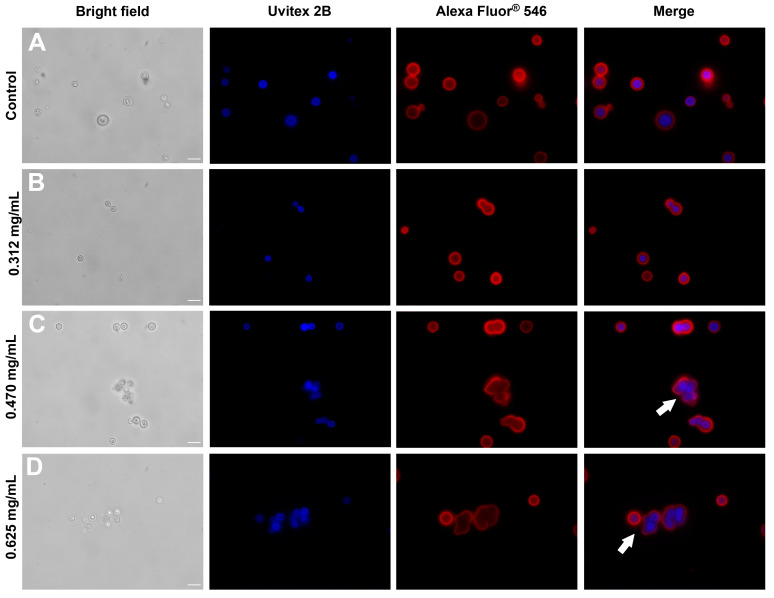
Optical fluorescence microscopy of *C. neoformans* in different concentrations of glatiramer acetate. First column, brightfield; second column, Uvitex 2B (cell wall markings in blue); third column, Alexa Fluor ® 546 (the polysaccharide capsule in red); and fourth column, overlapping images (Merge). (**A**) fungal cell control, (**B**) 0.312 mg/mL of GA, (**C**) 0.470 mg/mL of GA, and (**D**) 0.625 mg/mL of GA. White arrows indicate cell aggregation. Scale bar: 10 µm.

To investigate the impact of glatiramer acetate (GA) on the cell wall and the polysaccharide capsule of *C. neoformans*, the immunofluorescence technique was employed. Brightfield light optical microscopy revealed a consistent pattern of cellular aggregates, which was further confirmed at all GA concentrations (indicated by arrows in Figure 3C,D). This observation strongly suggests a direct relationship between the degree of aggregation/biofilm formation and the concentration of the drug. Notably, these cells appeared interconnected, exhibiting a unified polysaccharide capsule covering the entire surface of the conglomerate. While the capsule may exhibit structural variations, with the region adjacent to the cell wall (proximal) displaying a more cohesive polysaccharide mesh than the distal regions [52], it is evident that the labeling with the anti-GXM antibody Alexa Fluor^®^ 546 is discreet and diffused at concentrations of 0.470 mg/mL (Figure 3C) and 0.625 mg/mL (Figure 3D). This indicates that higher concentrations of GA influence the architecture of polysaccharides in the distal areas compared to the control (Figure 3A) and the condition of 0.312 mg/mL (Figure 3B). By utilizing immunofluorescence and carefully examining the resulting images, important insights into the structural modifications induced by GA on the cell wall and the polysaccharide capsule of *C. neoformans* were obtained. The observed patterns of cellular aggregates and changes in the polysaccharide architecture provide valuable evidence regarding the relationship between GA concentration and biofilm formation.

**Figure 4:**
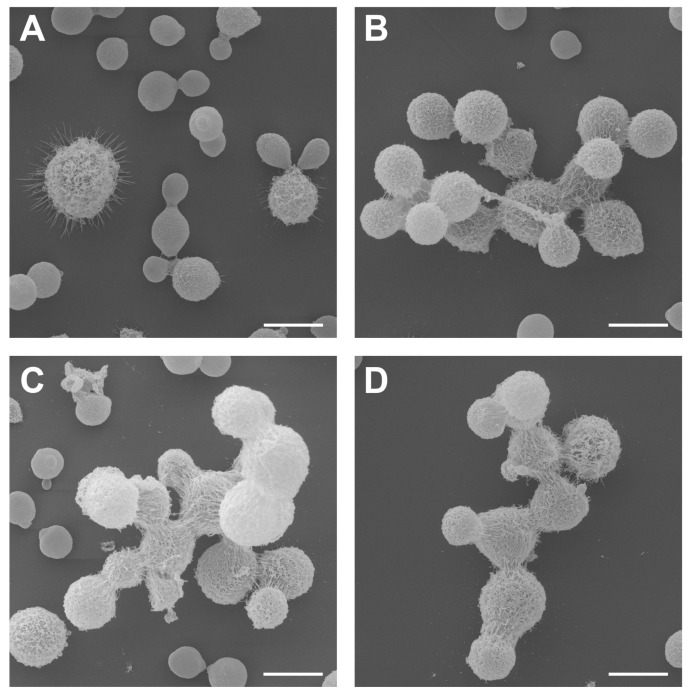
Scanning electron microscopy of *C. neoformans* cells under different concentrations of glatiramer acetate. (**A**) *C. neoformans* without drug (control), (**B**) *C. neoformans* cultured with 0.312 mg/mL of GA, (**C**) *C. neoformans* cultured with 0.470 mg/mL of GA, and (**D**) *C. neoformans* cultured with 0.625 mg/mL of GA. Scale bar: 5 µm.

By utilizing scanning electron microscopy, it was possible to analyze the aggregate cells of *C. neoformans* in the studied concentrations of glatiramer acetate. Reinforcing what had already been observed in previous microscopic analyses, all the micrographs were obtained from the conditions in which GA was present, as shown in the following: 0.312 mg/mL (Figure 4B); 0.470 mg/mL (Figure 4C); and 0.625 mg/mL (Figure 4D), where there was a marked cellular aggregation involving the formation of buds and the production of structures that can lead to biofilm formation. The cellular changes described were highlighted in relation to control fungal cell growth (Figure 4A), which showed cells more separated from each other and with natural processes of sprouts.

### 3.3. Analysis of the Physicochemical Properties of Polysaccharides Secreted (PS) by C. neoformans at Different Concentrations of Glatiramer Acetate

After studying the influence of glatiramer acetate at the cellular level and knowing that the polysaccharide capsule and the secreted one originates from different pools [51], the secreted polysaccharides (PS) of *C. neoformans* grown in different concentrations of GA was isolated to verify possible changes in their physicochemical properties.

**Figure 5:**
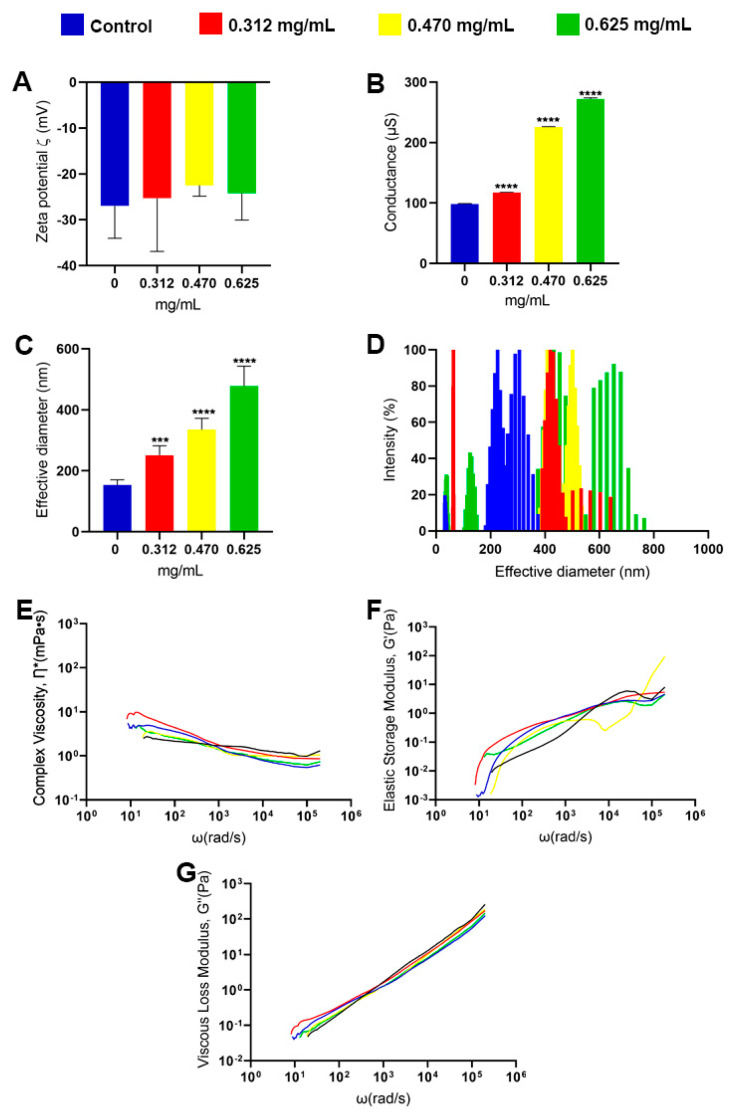
Analysis of physicochemical properties, size, and viscoelasticity of PS by *C. neoformans* in various concentrations of glatiramer acetate (GA). The control group was represented in blue, which did not receive any drug. The experimental groups were treated with varying concentrations of GA: red for 0.312 mg/mL, yellow for 0.470 mg/mL, and green for 0.625 mg/mL. (**A**) measurement of the Zeta potential, (**B**) measurement of conductance (µS), (**C**) effective diameter, (**D**) polydispersion of the size of PS, (**E**) measurement of the viscous complex Ƞ*(mPa•s), (**F**) measurement of the elastic modulus G’(Pa), and (**G**) viscous modulus G”(Pa) *** *p*-value < 0.001. **** *p*-value < 0.0001. Statistical treatment using the Student’s *t*-test.

The Zeta potential is a tool that allows us to measure changes and superficial effects that GA can generate in the secreted polysaccharides (PS) of *C. neoformans* in the different concentrations studied. Regarding the PS, there was no significant difference in the electronegativity between the conditions of the treatment with GA and the control (Figure 5A). However, the values obtained from the conductance measurements showed an increase in all the GA concentrations analyzed (Figure 5B), showing that there may be more ions adsorbed in the PS from the fungi treated.

Another crucial point to be verified is whether the secreted polysaccharides (PS) of *C. neoformans* had some structural change in relation to their size. Using the DLS as a study technique, it was possible to demonstrate that there was a gradual increase in the mean effective diameter of the PS at all concentrations of glatiramer acetate (0.312 mg/mL *p*-value < 0.001, 0.470 mg/mL and 0.625 mg/mL *p*-value < 0.0001, Student’s *t*-test) (Figure 5C). When analyzing the distribution of size in these PS, it was observed that the control (in blue) had a population around 50 nm and another with more intensity between 180 and 400 nm. Already in the lower concentration of GA (illustrated in red), there were two populations, one of which was around 50 nm and another with more intensity that varied between 400 and 600 nm. At the concentration of GA that is equivalent to the IC_50_ of the drug (in yellow), the same fiber profile can be observed, and at the highest concentration of GA (in green), three distinct populations were observed: one with about 50 nm, another more intermediary of 150 nm, and the other varying between 400 and 800 nm (Figure 5D). With the aid of techniques such as passive microrheology, it was observed that the PS of *C. neoformans* behaves like a viscoelastic liquid and that some factors, such as antibody binding, can change these properties [48]. For the analysis of the viscoelastic properties of PS in different concentrations of GA, the passive microrheology technique was used to obtain the modulus values of viscous (G’’), elastic (G’), and complex viscosity (η*). The results found for viscous complex (Figure 5E), elastic modulus (Figure 5F), and viscous modulus (Figure 5G) showed no changes between the analyses. As much as there seems to be some difference between the lines analyzed both in low and high frequencies, this difference is not maintained when the graph comes out from the logarithmic scale to a linear scale, indicating that the presence of the drug does not influence the viscoelastic properties of secreted PS.

## 4. Discussion

Glatiramer acetate (GA) was initially formulated to induce experimental autoimmune encephalitis (EAE, disease model for studying MS). However, to everyone’s surprise, GA prevented and suppressed the disease in the most varied animal species studied [53,54]. Copolymer 1 was synthesized with the aim of mimicking myelin basic protein (PMB) and thereby providing cross-reactivity with immune cells that react to the host itself [24]. Cross-reactivity has been demonstrated both by performing studies with B-cell monoclonal antibodies [23] and with T-cell-mediated cellular responses [55]. GA’s mechanism of action has not yet been fully elucidated, but it is known that its therapeutic action is related to immunomodulation at various levels of the immune system and a neuroprotective action, and this could be interesting during *C. neoformans*.

Knowing that the MIC corresponds to the lowest concentration of the drug that inhibits fungal cell growth [49], and with the visualization of the microdilution assay, it was possible to determine that the MIC of GA in *C. neoformans* cells was of 2.5 mg/mL. As there is no other pharmacological repositioning study involving GA and fungal cells, it is difficult to perform a comparison between the limited data already published in the literature. However, in studies involving bacterial models, the *Escherichia coli* species presented a GA MIC of 31 µg/mL, and in *Staphylococcus aureus*, 500 µg/mL [33]. The MIC value of *C. neoformans* is five times greater than the MIC value of the *S. aureus* bacteria, and this is probably due to the structural complexity that the fungal cell presents on its surface. The presence of virulence factors such as the polysaccharide capsule and melanin prevents drugs from easily accessing the fungal cell, requiring higher concentrations than usual in other species [56].

In the results of the microdilution assay (Figure 1A), it was verified that from the concentration of 2.5 mg/mL of GA, there was no more cell growth, and that in the concentrations of 0.156 mg/mL, 0.312 mg/mL, 0.625 mg/mL, and 1.250 mg/mL, it was possible to visualize colonies at the bottom of the microplate, but with reduced growth. For a drug to be classified as fungistatic, it must prevent the cell proliferation of fungal cells, and to be classified as a fungicide, it must prevent all fungal cells [57]. Corroborating the results found in the cell viability assay by XTT, it is possible to verify that GA has a fungistatic effect at its lowest concentrations of 0.156 mg/mL, 0.312 mg/mL, and IC_50_ 0.470 mg/mL, since even with a detectable reduction in cell proliferation in the microdilution assay, the cells remained viable. In addition, the concentration of 0.625 mg/mL of GA showed both a reduction in fungal proliferation and a significant decrease in cell viability when compared with the fungal growth control, indicating that after this concentration of GA, it started a fungicidal effect. Finally, the concentration of 1.250 mg/mL of GA showed existing colonies at the bottom of the plate, but according to what was found in the XTT result, there were no viable cells in this condition. The result found in the cell viability analysis indicated that up to the IC_50_ concentration of 0.470 mg/mL, GA has a fungistatic effect, and from the concentration of 0.625 mg/mL, it has a fungicidal effect.

The cell aggregation process may be an indication of possible biofilm formation, and the fact is that this process is being influenced by GA. Christiansen et al. [33], when studying the in vitro effect of this drug in some species of bacteria using the antimicrobial susceptibility test, were unable to determine the MIC for the Gram-negative bacterium *Pseudomonas aeruginosa* because GA induced a strong cell aggregation and the result was not possible to be analyzed by the software used. Both *P. aeruginosa* and *C. neoformans* are opportunistic pathogens that can form biofilms [58,59] and, although this comparison is made with 54 species of different microorganisms, both showed this same characteristic in vitro, evidencing the aggregating potential that GA plays [33].

This result found in measuring the size of the polysaccharide capsule showed that only the concentration of 0.625 mg/mL of GA led to a reduction in capsular size. However, when associated with the findings discussed in the cell viability studies by XTT, GA has fungicidal activity from the concentration of 0.625 mg/mL. Therefore, the reduction in capsular size may be associated with the presence of dead cells that had their development interrupted with reflections on the construction process of the polysaccharide capsule. The measurement of the cell body showed a significant increase only at the concentration of 0.470 mg/mL, indicating that this IC_50_ concentration may possibly stimulate *C. neoformans* cells to modulate a response to the host immune system, since the increase in the size of the cell body can influence phagocytosis escape and immune system evasion [5]. Referring once again to the results discussed in the XTT assay, the IC_50_ of 0.470 mg/mL is the highest concentration at which GA has a fungistatic effect, since after this concentration the fungicidal effect begins, and perhaps that is why the cell fungus presents this change in cell size, as it is reacting to GA which is preventing its development. This condition of cellular increase is only observed at 0.470 mg/mL because at the increased concentration of 0.625 mg/mL, the fungal cell already began losing its viability.

The concentration of 0.312 mg/mL of GA showed an increase in the Zeta potential (ζ) being more electronegative, suggesting that this increase is due to the increased exposure of glucuronic acid on the cell surface. This increase in the electronegativity may be related to a modulation that GA induces in the composition of the polysaccharide capsule, since the capsular size is only altered from a concentration of 0.625 mg/mL. In the case of conductance, it is possible to observe a decrease in all conditions in which the drug was present. GA has cationic charges that oppose the anionic charges generated by glucuronic acid on the cell surface. Therefore, it is plausible that the electrostatic interaction between these two components could have neutralized most of the ions that would be free both in the medium and in the cell, thus decreasing the conductivity.

According to the micrographs obtained, it is noticeable that the drug in question possibly causes a cellular alteration that generates several interconnected sprouts. Araújo et al. [60] characterized that through video microscopy studies in *C. Neoformans*, the budding of daughter cells always occurs in the same region of the mother-cell. That said, it is possible to identify that under the conditions in which the fungal cell has contact with GA, the process of the budding of daughter-cells occurs in two or more regions of the mother cell. A weakness in the detachment of daughter cells from their progenitors and a change in budding events were also perceived, but this observation needs to be better studied [60].

After studying the influence of glatiramer acetate at the cellular level and knowing that the polysaccharide capsule and the secreted one originates from different pools [51], the secreted polysaccharides (PS) of *C. neoformans* cultivated in different concentrations of GA were isolated to verify possible modifications in its physicochemical properties. Unlike what was observed in the analysis of the conductance of the cell surface of *C. neoformans*, in which there was a decrease in all concentrations of GA, with PS, the phenomenon observed is a gradual increase in conductance. A hypothesis to explain this difference may be that the fungal cell has a larger contact surface for this cationic polypeptide (GA) when compared to PS fibers. GA has a complex structure of amino acids whose molecular mass ranges from 5000 to 9000 Da [24] and that interacts electrostatically. Even the PS presenting the negative charge of the glucuronic acid in its composition seems to not have been sufficient to neutralize the GA charges. Therefore, we concluded that the load of the PS fibers per se do not present significant differences between the different samples; however, the global load is high as the drug concentration increases, even after consecutive washings with apyrogenic water, indicating that GA interacts “strongly” with the fibers.

The results found through the DLS demonstrated that the PS produced by *C. neoformans* gradually increased in size as the concentrations of GA increased: the PS control without the presence of the drug had a mean diameter of around 180 nm (±17.7), the PS in 0.312 mg/mL of GA had a mean diameter in the range of 300 nm (±27.9), while the PS in the presence of 0.470 mg/mL of GA had a mean diameter close to 400 nm (±33.5), and the PS in 0.625 mg/mL of GA had a mean diameter of 500 nm (±58.3). Previous results showed that the polysaccharide capsule did not increase in size in the concentrations of 0.312 mg/mL and 0.470 mg/mL; however, the PS in these conditions were higher than those in the control. The concentration of 0.625 mg/mL of GA, on the other hand, led to a decrease in the polysaccharide capsule, since in this condition, GA had a fungicidal effect and curiously showed the largest PS size. One possibility to explain this phenomenon is that *C. neoformans* cells, in reaction to the stressful environment containing this drug, begin to secrete larger polysaccharides to be used in the construction of the polysaccharide capsule; however, due to the observed action of decreasing the conductance of fungal cells, the GA can somehow impede the architecture of the capsule, causing the PS to be free in the medium. Much of the information known about the properties that the polysaccharide capsule has in the interaction with the host was obtained through studies with PS, so they may share the same functions [61]. *C. neoformans* manages to modulate the production of these polysaccharides to act as a form of defense [62], as observed in conditions with GA. A larger PS makes phagocytosis by macrophages even more difficult, allowing it to withstand certain forces that can be exerted and may even favor biofilm formation [61,63].

Based on the in vitro results, it was possible to conclude that glatiramer acetate (GA) is a possible candidate for pharmacological repositioning in the treatment of infections caused by *C. neoformans*. This drug has an IC_50_ of 0.470 mg/mL and an MIC of 2.5 mg/mL, having a fungistatic effect up to the IC_50_ concentration and a fungicidal effect from 0.625 mg/mL of GA. From the concentrations at which this drug appears as a fungicide, there was a decrease in the size of the polysaccharide capsule, which is an important parameter for future studies of pharmacological synergism. The conductance of fungal cells was decreased in the presence of this drug, while that of secreted polysaccharides was increased. To counteract the actions induced by GA, several notable effects were observed, including increased cell aggregation, potential stimulation of biofilm formation, disruption of the budding process, and an augmentation in the size of secreted polysaccharides across all conditions tested for the drug. These observations indicate alterations in virulence factors.

## Data Availability

The data presented in this study are available within the article.
